# Challenges in measurement of adolescent mental health: how are gender patterns affected when level of symptoms is analysed simultaneously with impairment?

**DOI:** 10.1186/s12874-024-02385-1

**Published:** 2024-11-07

**Authors:** Curt Hagquist

**Affiliations:** Department of Education and Special Education, University of Gothenburg, Box 300, Gothenburg, 405 30 Sweden

**Keywords:** Adolescents, Mental health, Psychosomatic symptoms, Impairment, Measurement, Gender

## Abstract

**Background:**

Adolescent mental health surveys in public health are sometimes questioned because of their main focus on self-reported symptoms, lacking data on impairment, e.g. the consequences on everyday life of the mental health problems. While public health studies typically reveal higher prevalence rates of internalising problems for girls than boys, there are indications that the gender pattern may change when self-reported data on symptoms are analysed simultaneously with impairment. The purpose is to determine how gender patterns of adolescent mental health solely based on symptoms are affected when level of symptoms is analysed simultaneously with impairment.

**Methods:**

Questionnaire data on adolescent mental health were collected in schools by Statistics Sweden in the autumn of 2009 as part of a national total population study in grades 6 and 9 in Sweden. In this study only data from grade 9 students are used (*n* = 91 627; response rate = 80 per cent). Psychosomatic symptoms were measured with the Psychosomatic Problems scale including eight items. Impairment was measured with four items included in the SDQ impact supplement. The associations between these key constructs were analysed with logistic regression and contingency tables.

**Results:**

When analysing variables on psychosomatic symptoms and impairment independently, the results are consistent with typical findings of gender patterns in adolescent internalising mental health. Girls report both more psychosomatic symptoms, and more negative consequences in everyday life, than boys. The gender patterns are, however, strongly affected when impairment is conditioned on level of psychosomatic symptoms. Except for the Home Life setting, in the settings of Friendships, Classroom Learning and Leisure Activities, the previously reported gender pattern favoring higher disturbances among girls becomes partly reversed implying that boys report more negative consequences than girls. Hence, while girls report a higher prevalence of psychosomatic symptoms, boys appear to suffer from such symptoms more than, or as much as, girls in three out of four everyday life settings.

**Conclusions:**

The study confirms the insufficiency of solely including data on symptoms in the measurement of adolescent mental health. Regardless of the causes of the complex gender pattern shown in this study, the results highlight the importance of simultaneous inclusion of indicators of impairment as well as symptom counts and frequency in the measurement of adolescent mental health.

## Background

There are plenty of empirical studies indicating that adolescent mental health has deteriorated in many Western countries during the last few decades [1]. These studies are commonly based on self-reports using questionnaires. A typical example is the WHO collaborative Health Behaviour in School-aged Children (HBSC) study with recurrent data collections every fourth year among students 11, 13 and 15 years old [2]. The HBSC study, which started in the 1980’s, currently includes more than 50 countries in Europe and North America. The outcomes from the HBSC study are disseminated by WHO, OECD and other international organisations, as well as frequently used for research. Reports based on the Swedish HBSC data show that not just the prevalence rates of internalising mental health problems are higher among girls than boys, but also that the gender gap has increased over time [3]. The most recent HBSC data collected in Sweden 2021/22 show the highest figures of subjective health complaints at ages 13 and 15 since the start of the study in the 1980s [4]. The credibility and validity of the HBSC study has been questioned because of its methodology and measurement of mental health [5]. In particular, this applies to the trend reports on increasing mental health problems among young people. In a recent study based on HBSC data, Högberg et al. [6] rejected some of the criticism by demonstrating that reports about increasing complaints in adolescents “…*cannot be explained as being primarily due to a greater inclination to report trivial complaints.*” (p.625). Another point of criticism concerns that public health surveys are mainly based on questions about symptoms, not including questions about impairment, e.g. the consequences on everyday life of mental health symptoms. Although the family and school contexts are of paramount importance for young people’s growing up conditions, the impacts of mental health problems in these areas are not directly addressed in the HBSC survey questionnaires [2].

One of the more outspoken critics of the lack of impairment data in child and adolescent mental health research, the Dutch psychiatrist and epidemiologist Frank Verhulst, question “*…to what extent changes in symptom prevalence over time are accompanied by changes in impairment*.”, concluding that “*In order to evaluate increases in problems such as anxiety and depression symptoms in children and adolescents*,* information on impairment in everyday functioning and of other indicators of child and adolescent well-being are crucial*.” (p. 395) [7].

In contrast to the field of public health, in child and adolescent psychiatry it is more common to focus on both symptoms and impairment. A popular measurement instrument in child and adolescent mental health epidemiology, the Strength and Difficulties Questionnaire (SDQ), includes symptoms scales as well as an impact scale [8], but surprisingly few SDQ studies have included impact data. Using SDQ data in a German sample, Wille et al. [9] concluded that data on impairment help to identify groups of adolescents at high risk who previously not were detected.

In clinical settings, inquiries about impairment is a way to examine the clinical significance of symptoms [10]. While the approach is reflected in the Diagnostic and statistical manual of mental disorders developed by the American Psychiatric Association (APA) [11], the use of impairment as a criteria for diagnoses has been criticised [12].

Reviewing the literature over 40 years, Roberts et al. [13] reported that adjustment for impairment implied lower prevalence rates. More recent epidemiological studies indicate that inclusion of impairment for classification of mental disorders may affect the prevalence for some diagnoses [10], but making no difference for others, e.g. depression [12].

The potential impact of inclusion of indicators of functional impairment in trend analyses has been investigated in a Swedish study based on questionnaire data collected 1988–2011 [14]. This study showed that the prevalence rates became lower when indicators of functional impairment were combined with psychosomatic problems, but that the shape of the trend curves remained about the same.

Another related, but less highlighted, measurement issue is whether gender patterns of prevalence are changed when data about impairment are considered simultaneously with symptoms of mental ill health. There are some studies demonstrating the importance of considering impairment in order to understand and interpret gender differences in symptoms of mental health. For example, a couple of Norwegian studies have shown that the psychosocial functioning among boys was more negatively affected by symptoms of anxiety and depression than among girls [15, 16].

## Methods

### Aim

The aim of the study is to determine how gender patterns of adolescent mental health solely based on symptoms are affected when level of symptoms is analysed simultaneously with impairment.

### Data collection

Data were collected by Statistics Sweden in the autumn of 2009 as part of a nationwide survey on adolescent mental health in Sweden. All students in Sweden in grade 6 (~ 12 years old) and grade 9 (~ 15 years old) comprised the target group, in all 207,700 students. For the purpose of this study only data from grade 9 students are used (*n* = 91 627; response rate = 80 per cent).

The data were collected in schools with questionnaires which were distributed to the students by persons working on behalf of Statistics Sweden. Students were informed that participation was voluntary.

The data were provided by the Swedish National Board of Health and Welfare, based on an ethical application approved by the Central Ethical Review Board in Sweden (Ö 39–2011).

### Variables and measures

The nationwide survey questionnaire [17] included three major instruments: The Kidscreen questionnaire on health-related quality of life [18], the Strength and Difficulties Questionnaire (SDQ) [8, 19] and the Psychosomatic Problems (PSP)-Scale [20]. In the current study, dichotomised PSP data are used as well as SDQ data on impairment and item scales on child-parent relations and economic resources based on Kidscreen data.

#### Impairment

Four areas of impairment were assessed which represent key domains in constituting everyday life of young people, covering both school and leisure hours, as well as both family and peer relations.

From the SDQ impact supplement two main questions were retrieved:

- “Overall, do you think that you have difficulties in one or more of the following areas: emotions, concentration, behaviour or being able to get on with other people?”

There were four response alternatives: No; Yes minor difficulties; Yes, definite difficulties; Yes, severe difficulties. This question was also used as a filter to route the participants. Those responding to a Yes alternative (*n* = 33711) were directed to additional questions, including:

- “Do the difficulties interfere with your everyday life in the following areas? Home Life; Friendships; Classroom Learning; Leisure Activities”.

There were four response alternatives: Not at all; Only a little; Quite a lot; A great deal. In the analyses, the four variables were dichotomised by collapsing the first and second response categories and the third and fourth categories.

#### The psychosomatic problems (PSP) scale

The PSP-scale was used as the measure of mental health because many large-scale studies in public health are using psychosomatic complaints to tap information about adolescent mental health. The PSP scale consists of eight items: ‘had difficulty in concentrating’, ‘had difficulty in sleeping’, ‘suffered from headaches’, ‘suffered from stomach aches’, ‘felt tense’, ‘had little appetite’, ‘felt sad’ and ‘felt giddy’. The response categories for all of these items are: never, seldom, sometimes, often and always. The categories are ordered in terms of implied frequency: the greater the frequency, the lower the adolescents’ well-being.

The PSP scale shares many similarities with the psychosomatic complaints scale used in the WHO collaborative study on Health Behaviour in School-aged Children (HBSC) (Inchley, 2020). The PSP scale shows sound psychometric properties, the items work invariantly along the latent variable and the item categories appear in a natural order, which enables the responses of the students to be properly summarised and distributed across the latent variable [20].

The conceptualisation, construction and psychometric properties of the PSP scale are described by Hagquist [20].

#### Additional scales and variables

Questions from the Kidscreen questionnaire were used to construct a composite measure of child-parent relations (the Child-Parent Relations Scale) and a composite measure of economic resources (the Perceived Economic Resources Scale). The construction and psychometric properties of these scales are described by Hagquist [21]. In addition, variables on gender, country of birth and type of family residency were also included in the multivariate analyses [21].

### Analysis

The psychometric properties of the PSP-scale were analysed with Rasch Measurement Theory [22, 23]. A key purpose of applying Rasch analysis was to examine if the current data met measurement requirements for constructions of scales by summation of the person’s responses to individual items. Three key aspects were examined:

*Invariance, which is an integral part of the Rasch model. Statistical tests indicate whether the the empirical data fit the Rasch model or not.

*The ordering of the response categories, i.e. if the item thresholds appear in a natural order or not.

* The targeting of the items to the population that was subjected to the analysis, i.e. if the severity of the items matches the distribution of the persons’ psychosomatic problems.

As part of the Rasch analysis, the total raw scores were nonlinear transformed to a scale of logit values. If the items work invariantly, complete data are not required, i.e. logit values are also provided for persons who have not responded on all items [24, 25].

The PSP-scale was dichotomised, based on the cut-off point for the 90th percentile for the entire sample, representing higher degree of psychosomatic problems. The proportion of boys and girls on or above that cut-off point was 5 and 16 per cent respectively.

Contingency table analysis and multivariate logistic regression were applied for analysis of the associations between psychosomatic symptoms and impairment. Students reporting no mental health difficulties at all and therefore not answering the four sub questions about impact of difficulties were coded “Not at all” in the variables for the four sub questions.

Multivariate logistic regression analysis was applied, separately for boys and girls. In each of the four sub settings (Home Life; Friendships; Classroom Learning; Leisure Activities), a variable on experiences of impairment (the dependent variable) was regressed on the PSP variable, and four additional independent variables (type of family residency, country of birth, economic resources and child-parent relations).

The Rasch psychometric analysis of the PSP scale was performed with the software RUMM2030Plus [26].

## Results

Overall, the PSP-scale met the requirements of the Rasch model well, including proper ordering of the response categories. The person separation index, a Cronbach’s alpha analogue, showed a value of 0.838 (*n* = 89629). A couple of items indicated Differential Item Functioning (DIF) across genders. Considering the trade-off between validity and fit to the Rasch model [27], these items were neither removed nor resolved for DIF.

In the entire sample 64% of the boys and 54% of the girls reported no difficulties in any of the following areas: “emotions, concentration, behaviour or being able to get on with other people”. The corresponding proportions (boys/girls) for minor difficulties were 24/34; for definite difficulties 5/7 and for severe difficulties 2/2.

**Fig. 1:**
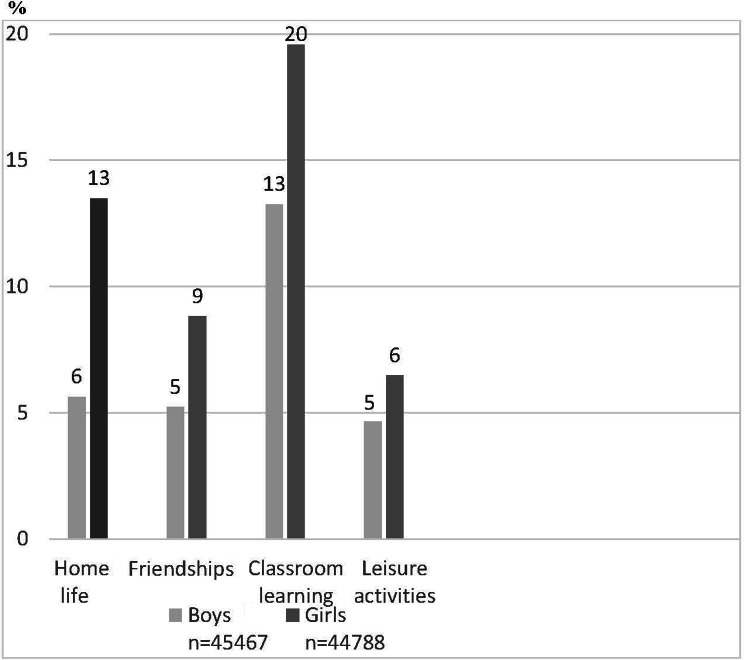
Responses to the question “*Do the difficulties interfere with your everyday life in the following areas?* Proportions of responses of Quite a lot or A great deal among boys and girls

In Fig. 1 the proportion of boys and girls in grade 9 who report that the difficulties with emotions, concentration, behaviour or being able to get on with other people interfere with their everyday life Quite a lot or A great deal in four areas are shown.

Figure 1 shows that in all four settings the proportions of girls who report Quite a lot or A great deal of difficulties are higher than for boys. The highest proportions are for classroom learning and home life. 20 per cent of the girls and 13 per cent of the boys reported mental health problems that had a negative impact on their schoolwork. The corresponding proportions for a negative impact on the family life are 13 and 6 per cent respectively.

**Fig. 2:**
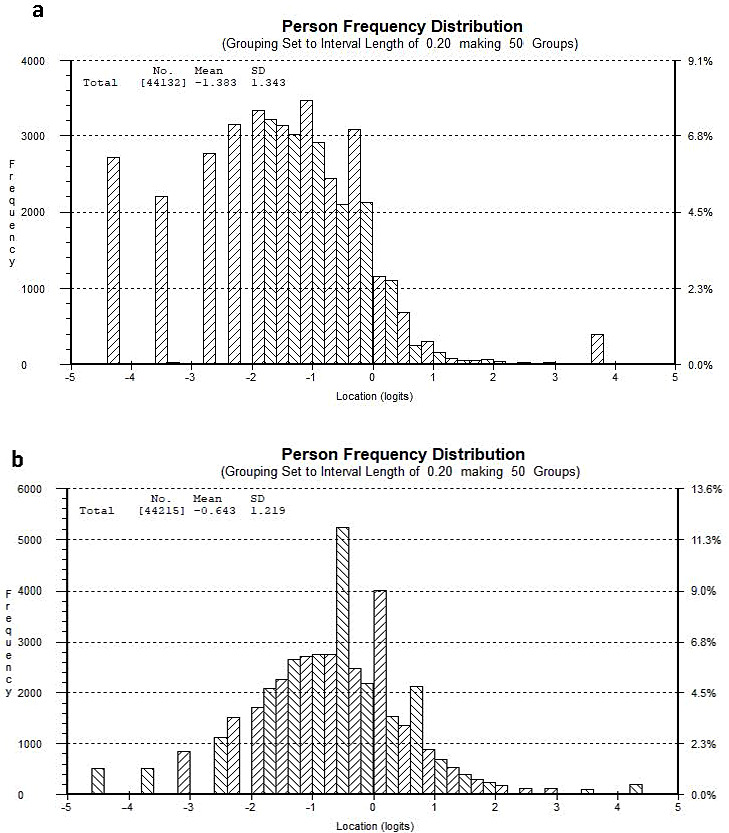
(**a**) Person frequency distribution of logit values of the PSP scale for boys. The higher the values (logits), the higher degree of psychosomatic problems. (**b**) Person frequency distribution of logits values of the PSP scale for girls. The higher the values (logits), the higher degree of psychosomatic problems

In Fig. 2a and b the grade 9 students’ frequency distribution of psychosomatic problems is shown for boys and girls respectively.

Figure 2a and b shows that the population of boys is distributed more to the left along the latent variable while the population of girls is distributed closer to the middle of scale compared to boys. This gender difference is reflected by higher mean values for girls than boys, revealing that girls on average experience psychosomatic problems to a higher degree than boys do.

**Fig. 3:**
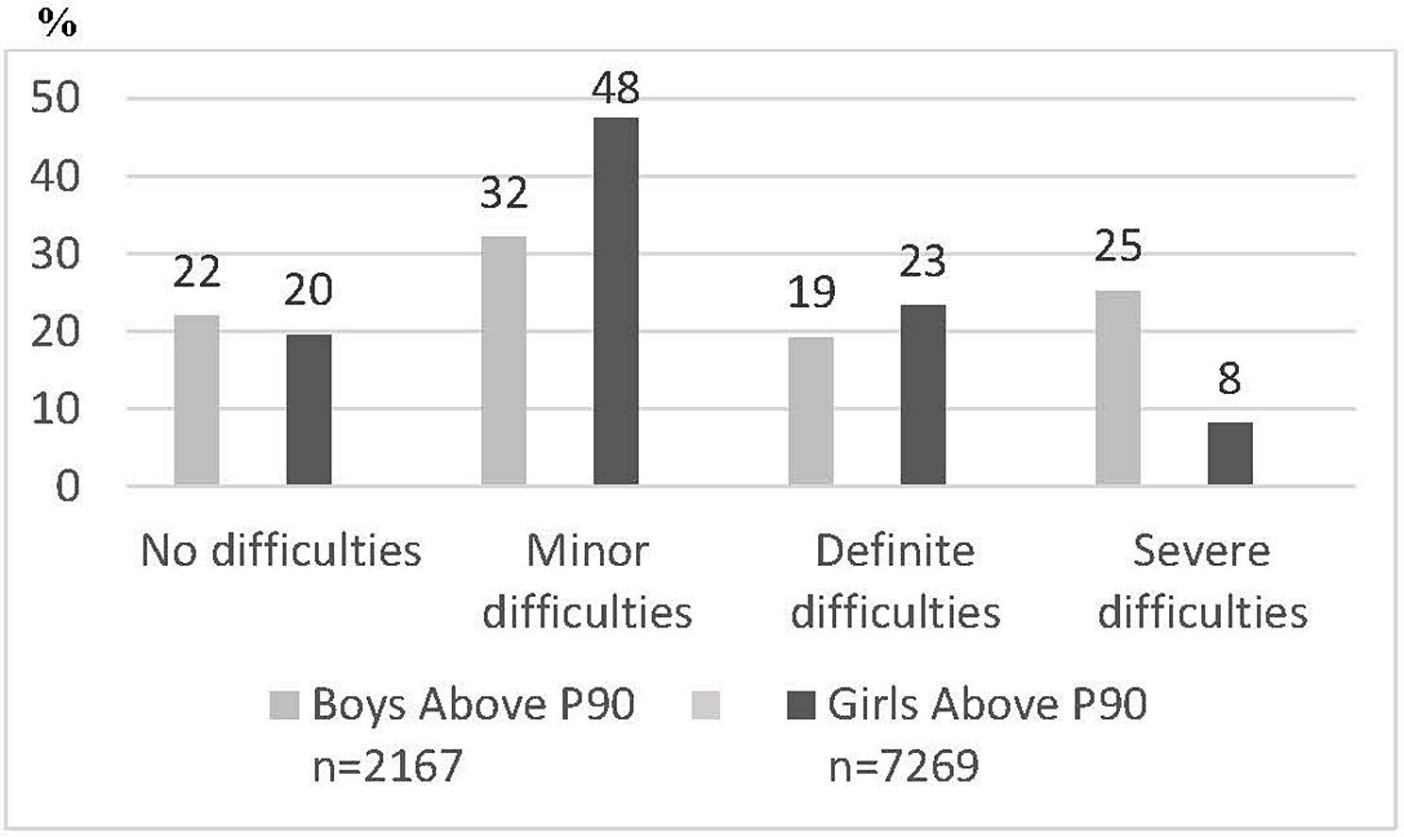
Proportion of boys and girls with no difficulties, minor difficulties, definite difficulties and severe difficulties with emotions, concentration, behaviour or being able to get on with other people, among those with higher degree of psychosomatic problems (above Perc 90)

In Fig. 3 the proportions of boys and girls in grade 9 with difficulties in one or more of the areas of emotions, concentration, behaviour or being able to get on with other people are shown for boys and girls with higher degree of psychosomatic symptoms.

Figure 3 shows that among students with higher degree of psychosomatic symptoms, girls report minor and definite difficulties more frequently than boys, while the proportion of students who report severe difficulties is three times higher among boys than girls.

**Fig. 4:**
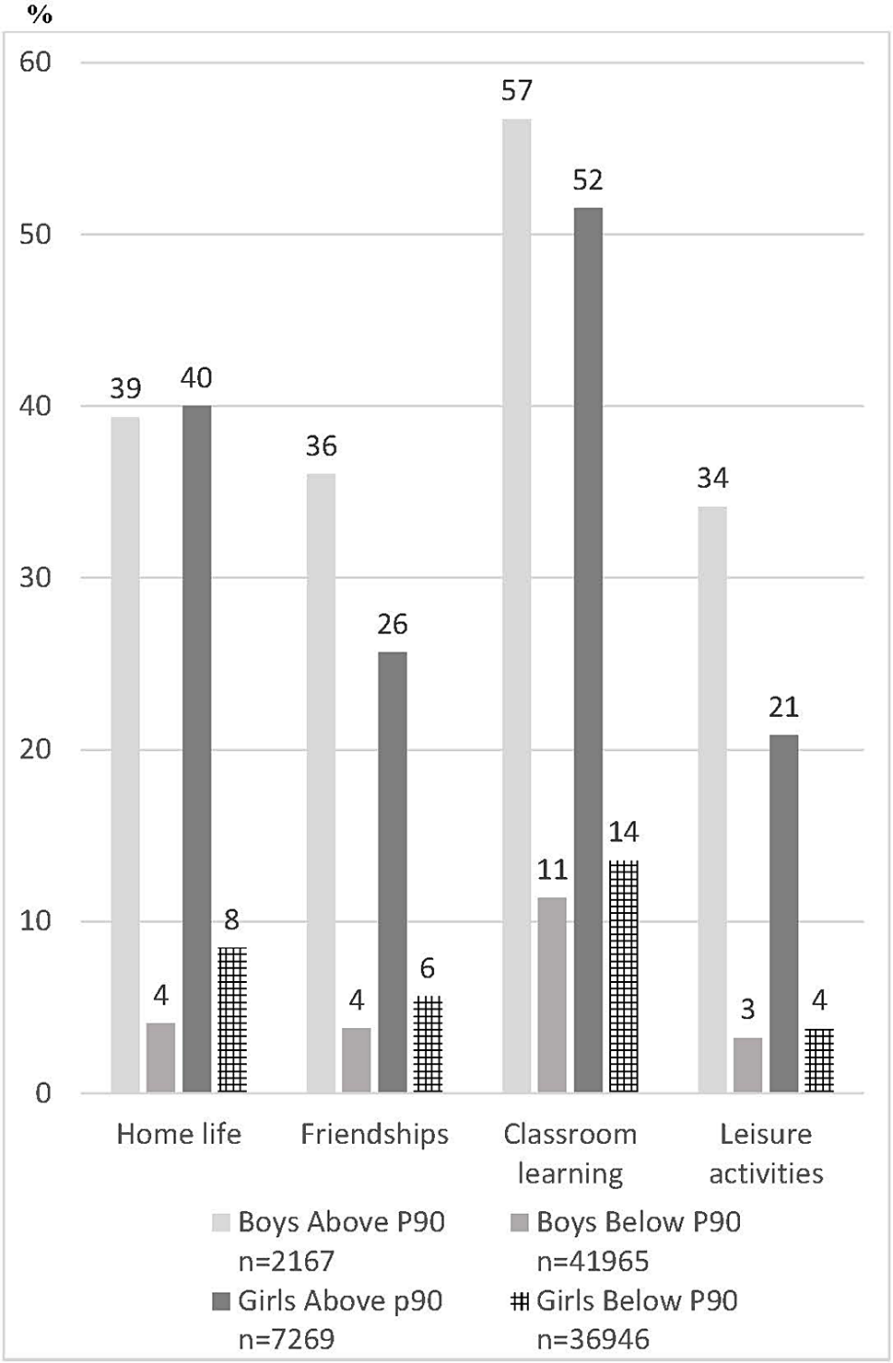
Proportion of boys and girls in grade 9 with difficulties (quite a lot or a great deal) with emotions, concentration, behaviour or being able to get on with other people, in different areas among those with higher degrees of psychosomatic problems (above Perc 90) and among those with lower degrees (below Perc 90) of psychosomatic problems respectively

In Fig. 4 the proportions of boys and girls in grade 9 with quite a lot or a great deal with difficulties with emotions, concentration, behaviour or being able to get on with other people in different areas are shown, among those with higher and lower degree of psychosomatic problems respectively.

Figure 4 shows that there is a strong relationship between psychosomatic symptoms and consequences of mental health problems on everyday life in all four settings. Figure 4 also shows that the gender patterns reported in Fig. 1 are drastically changed when impairment is conditioned on the degree of psychosomatic symptoms. In the group of students reporting a higher degree of psychosomatic symptoms, the proportion of boys reporting negative consequences is as big as among girls in one setting and higher than girls in the other three settings. The strong association between psychosomatic symptoms and impairment that is evident in Fig. 4 is confirmed by multivariate binary logistic regression. The estimated odds ratios (not shown in a table) are high in all four impairment settings, in particular among boys. The odds for difficulties in leisure activities among boys is about nine times higher among those with higher degree of psychosomatic symptoms (> P90) compared to those with lower degree (< P90). For Home life and Friendship among boys, the corresponding odds were about eight time higher and for Classroom learning about seven times. Among girls, the corresponding odds were about four to five times higher in the four settings comparing those with higher degree of psychosomatic symptoms with those with lower degree. Likelihood Ratio tests based on a pooled data set of boys and girls confirmed that the associations between psychosomatic symptoms and impairment were moderated by gender.

**Fig. 5:**
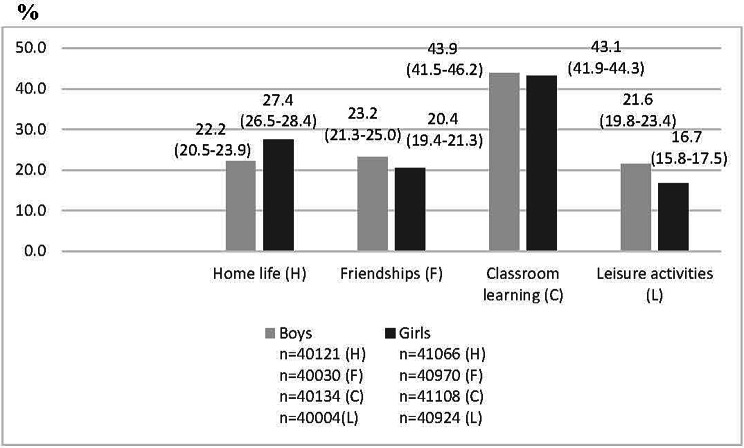
Predictive percentages of impairment (quite a lot or a great deal) among boys and girls in grade 9 in four different areas, controlling for psychosomatic problems and background variables. 95% Confidence Intervals

In Fig. 5, the outcomes from the multivariate logistic binary regression analyses among boys and girls in grade 9 are reported as predicted percentages regressing experiences of impairment (difficulties with emotions, concentration, behaviour or being able to get on with other people), in four settings on psychosomatic symptoms, simultaneously controlling for type of family residency, country of birth, economic resources and child-parents relations.

Figure 5 indicates that there are some changes in the gender patterns compared to the bivariate contingency table analysis (Fig. 4) when a multivariate approach is applied for the analysis of the associations between psychosomatic symptoms and impairment. The predictive values for quite a lot or a great deal of difficulties for home life become higher for girls than for boys and only slightly higher for boys than for girls on classroom learning. The patterns for the friendships and leisure activities settings indicate higher predictive values for boys than girls, but the gender differences are less pronounced compared to those reported in the bivariate contingency table analysis (Fig. 4).

## Discussion

This study shows that girls report more psychosomatic symptoms than boys, and that girls report more functional impact on their everyday life from mental health problems when these two types of variables are analysed independently of each other. However, these gender patterns are altered when functional impairment is conditioned on high levels of reported psychosomatic symptoms. In three out of four settings, the previously described gender pattern is reversed in the unadjusted analysis, and in one setting, the rates of impairment become equal for boys and girls. Broadly, this pattern holds even in multivariate analyses, regressing impairment on psychosomatic symptoms while controlling for socio-demographic factors and child-parent relations. Only in the home life setting, are the predictive percentages for impairment higher for girls than boys.

The prevalence rates reported here for symptoms and impairment variables, when independently estimated, conform to the typical gender patterns of internalising mental health problems occurring in a large number of countries around the world [28]. In contrast, however, these gender patterns are changed when the commonly used approach solely based on measurement of symptoms is broaden by simultaneously analysing measures of impairment. A substantive interpretation of the results would be that boys suffer from symptoms as much as, or more than, girls in three out of four everyday life settings, while girls are reporting psychosomatic symptoms more frequently. This interpretation is partly in agreement with the Norwegian cross-sectional and longitudinal studies referenced in the introduction section of this paper, which showed that gender moderated the association between anxiety and depression, and impairment [15, 16]. Interpreting their results, Derdikman et al. [16] refer to the gender paradox of co-morbidities reviewed and described by Eme [29], which stipulates that the gender showing the lowest prevalence of a disorder is the one more severely affected because of different thresholds.

## Conclusions

The current study nuances the results from previous studies solely based on self-reported internalising mental health symptoms by distinguishing between impairment in different settings associated with level of symptoms – and it does so for both boys and girls. There are a number of questions that need to be further examined in order to understand and interpret the complex gender patterns reported in the current study. Some of these questions concern methodological issues, e.g. whether girls are more prone to admit symptoms, and boys less prone because of different attitudes or thresholds. It would also be desirable to conduct psychometric analyses of potential differential item functioning (DIF) [25] across genders and at different levels of the impairment questions in the measurement of psychosomatic symptoms. Other issues concern the more substantive causes of a potential gender paradox, e.g. whether girls acquire better abilities to cope with symptoms than boys, or seeking professional help or support from friends to a larger extent than boys do. Regardless of the causes of the complex gender pattern shown in this study, the results confirm the importance of simultaneous inclusion of indicators of impairment as well as symptom counts and frequency in the measurement of adolescent mental health. In the light of the before mentioned questioning of the reliability of studies solely based on self-reported symptoms, the dual-factor approach is of particular value in trend and gender studies of adolescent mental health.

### Strengths and weaknesses

The study is based on a large number of students and the participation rate is high which enables robust analyses. In order to measure a construct, multiple items are usually preferable to single items because of higher reliability and validity. The psychosomatic problems scale and the control variables in the logistic regression analysis meet that request, while impairment is measured by single variables. Another limitation of the study is that it only focuses one dimension of mental health, psychosomatic problems.

## Data Availability

Request for data should be directed to the Swedish National Board of Health and Welfare, The data were collected in schools with questionnaires which were distributed to the students by persons working on behalf of Statistics Sweden. The students were informed orally and on the questionnaire that participation was voluntary. In addition, the students were informed that the answers they provided were protected by the Secrecy Act and the Personal Data Act. According to Swedish law, young people aged 15–17 give their own consent if they understand what the research means for themselves personally [https://etikprovningsmyndigheten.se/en/what-the-act-says/].
